# A framework for non-preserved consensus gene module detection in Johne's disease

**DOI:** 10.3389/fvets.2022.974444

**Published:** 2022-07-27

**Authors:** Maryam Heidari, Abbas Pakdel, Mohammad Reza Bakhtiarizadeh, Fariba Dehghanian

**Affiliations:** ^1^Department of Animal Science, College of Agriculture, Isfahan University of Technology, Isfahan, Iran; ^2^Department of Animal and Poultry Science, College of Aburaihan, University of Tehran, Tehran, Iran; ^3^Department of Biology, University of Isfahan, Isfahan, Iran

**Keywords:** Johne's disease, consensus modules, lncRNA-mRNA-TF networks, PPIs networks, RNA-Seq, hub genes

## Abstract

Johne's disease caused by Mycobacterium avium subsp. paratuberculosis (MAP) is a major concern in dairy industry. Since, the pathogenesis of the disease is not clearly known, it is necessary to develop an approach to discover molecular mechanisms behind this disease with high confidence. Biological studies often suffer from issues with reproducibility. Lack of a method to find stable modules in co-expression networks from different datasets related to Johne's disease motivated us to present a computational pipeline to identify non-preserved consensus modules. Two RNA-Seq datasets related to MAP infection were analyzed, and consensus modules were detected and were subjected to the preservation analysis. The non-preserved consensus modules in both datasets were determined as they are modules whose connectivity and density are affected by the disease. Long non-coding RNAs (lncRNAs) and TF genes in the non-preserved consensus modules were identified to construct integrated networks of lncRNA-mRNA-TF. These networks were confirmed by protein-protein interactions (PPIs) networks. Also, the overlapped hub genes between two datasets were considered hub genes of the consensus modules. Out of 66 consensus modules, 21 modules were non-preserved consensus modules, which were common in both datasets and 619 hub genes were members of these modules. Moreover, 34 lncRNA and 152 TF genes were identified in 12 and 19 non-preserved consensus modules, respectively. The predicted PPIs in 17 non-preserved consensus modules were significant, and 283 hub genes were commonly identified in both co-expression and PPIs networks. Functional enrichment analysis revealed that eight out of 21 modules were significantly enriched for biological processes associated with Johne's disease including “inflammatory response,” “interleukin-1-mediated signaling pathway”, “type I interferon signaling pathway,” “cytokine-mediated signaling pathway,” “regulation of interferon-beta production,” and “response to interferon-gamma.” Moreover, some genes (hub mRNA, TF, and lncRNA) were introduced as potential candidates for Johne's disease pathogenesis such as *TLR2, NFKB1, IRF1, ATF3, TREM1, CDH26, HMGB1, STAT1, ISG15, CASP3*. This study expanded our knowledge of molecular mechanisms involved in Johne's disease, and the presented pipeline enabled us to achieve more valid results.

## Introduction

Johne's disease (JD) is one of the most serious chronic infectious diseases of ruminants worldwide. *Mycobacterium avium subsp. paratuberculosis* (MAP) is the causative agent of the disease. JD causes significant economic losses due to the symptoms and problems including diarrhea, weight loss, decreased milk production, premature culling, decreased productive life and increased mortality (1–3). Animals usually get infected in the first months of their life through the fecal-oral route or ingestion of infected colostrum or milk; however, infection *in utero* is also reported (4–6). After entering the animal's cell, MAP is phagocytized by intestinal macrophages, which play a substantial role in beginning the appropriate innate and adaptive immune response (7). Infected macrophages secrete inflammatory cytokines such as TNF-α, IL-10, and IL-12 and trigger the adaptive immune response through the production of gamma interferon (IFNγ) (8, 9). The main characteristic of host immunity to MAP infection contains early Th1 response (pro-inflammatory and cytotoxic response) that finally changes to Th2 response (antibody-based response) (9). Most of the time, MAP can escape the immune response and survive and proliferate within phagosomes. The main problem that delays the diagnosis of MAP infection is the long incubation period during which the infection can be spread across the herd (10). Hence, it is necessary to facilitate early diagnosis of this disease by uncovering the underlying molecular mechanisms. Different approaches have been applied to address this issue. A great number of studies have been conducted with the purpose of finding differentially expressed genes (11–18). Park et al. (18) performed gene expression analysis of immune regulatory genes during MAP infection in the whole blood of cattle. According to their results, downregulation and upregulation of these genes were indicative of suppression of the Th1 response due to MAP infection, loss of granuloma integrity, and finally enhanced survival of MAP during subclinical stages (18). In a more recent study, Ariel et al. identified a considerable number of differentially expressed genes in JD negative macrophages. They reported that some pathways such as energy production pathways and lipid homeostasis were affected by MAP, in addition to immune pathways (17). Researchers in several studies tried to identify genomic regions associated with JD (19–25). Mallikarjunappa et al. performed a genome-wide association study of previously analyzed 50K SNP-chip. They could validate previous findings and identify new QTL associated with the MAP infection on 15, 16, 20, and 21 bovine chromosomes (22). McGovern et al. found putative MAP susceptibility QTLs on several bovine chromosomes and reported some functional candidate genes (20).

Since genes are co-expressed during the processes of disease development, they probably co-regulate biological processes or functions across the processes (26–28). Therefore, gene co-expression network analysis, as a complement of traditional differential gene expression analysis, is a powerful system biology approach to discover new gene functions and regulatory relationships involved in diseases like JD. These networks provide the possibility to systematically identify co-expressed genes, called module (29). According to the assumption behind this analysis, highly connected genes within each module are functionally coherent and exhibit similar biological relationships across different stages or conditions (26). Ibeagha-Awemu et al. (2018) used weighted gene co-expression network (WGCNA) and functional enrichment analyses to identify important genes, pathways, and TFs regulating MAP infection. They found *CTSH* and *MERTK* hub genes play a part in the degradation of lysosomal proteins and phagocytosis of apoptotic cells, respectively. Moreover, *SPI1* and *EP300* were the most significantly enriched TFs in the co-expressed modules related to JD (30). In our previous study to better understand the underlying molecular mechanisms regulating JD, WGCNA was applied, and integrated networks including long non-coding RNAs (lncRNAs), mRNA and transcription factors (TFs) were constructed. As a result, several genes potentially associated with MAP infection were identified including *SLC11A1, MAPK8IP1, HMGCR, IFNGR1, CMPK2, CORO1A, IRF1, LDLR, BOLA-DMB*, and *BOLA-DMA* (28).

A big concern related to findings from biological studies, especially co-expression analysis, is the reproducibility of the results when tested in independent data. In other words, one important question is how the identified modules are sensitive to the input datasets, or stability. Stable modules are comprised of tightly co-expressed genes that co-occur in networks inferred from different datasets and can be used to explore core components of the biological processes of interest. It is supposed that these modules are not affected by specific batch or experimental artifacts. In this regard, all the previous studies used only one dataset to construct gene co-expression networks, while the identified modules may change in another dataset. Compared to module detection from a single data, stable module identification approach, called consensus modules, can improve reproducibility by integrating data from several studies. In spite of the limited studies investigating co-expression network analysis in JD, there are no studies to construct consensus modules in this disease across different datasets. In this study, for the first time, a computational pipeline was developed to identify non-preserved consensus modules associated with JD, which enable us to explore the potential molecular mechanisms of this disorder. To this end, the consensus modules were constructed across two datasets, based on their normal samples. Then, preservation of the consensus modules was assessed in infected samples of both datasets and the non-preserved modules in both datasets were considered as non-preserved consensus modules and were followed by functional enrichment analysis to explore the biological function of the important modules and their potential to be involved in JD. Accordingly, an integrated gene regulatory network was constructed, which provides novel insights into the pathological process of this disease.

## Materials and methods

### Datasets

Two RNA-Seq datasets related to MAP infection were considered for this study. Both were obtained from the publicly available gene expression omnibus (GEO) database of the National Center for Biotechnology Research (NCBI). The first dataset (accession number GSE62048) contained 35 monocyte-derived macrophages (MDM) samples collected from seven Holstein-Friesian cows and were infected *in vitro* with a clinical isolate of MAP. There were two groups of samples including 21 control (at 0, 2, and 6 h after infection with MAP) and 14 infected (at 2 and 6 h after infection with MAP) (31). The second one (accession number GSE98363) comprised 72 monocyte-derived macrophages samples from 12 dairy cows. Animals were divided into two groups of positive and negative according to disease status so that each group includes six cows. For each cow, monocyte-derived macrophages were cultured and exposed *ex vivo* to MAP infection. The samples were harvested at 4 and 24 h after infection (24 samples) for the control group and 1, 4, 8, and 24 h after infection (48 samples) for the infected group (17). Samples in both datasets were sequenced using Illumina HiSeq 2000, while the first dataset was single-end and the second one was paired-end.

### RNA-Seq data analysis

For both datasets, the quality of the raw reads was checked using FastQC software (version 0.11.5) (32). After that, trimming was done to remove the low-quality reads using Trimmomatic software v0.38 (33). Then, the clean reads were aligned to the bovine reference genome (ARS-UCD1.2 from ENSEMBL database) using Hisat2 software (version 2.0.4) (34). The read counts per annotated gene were generated using Htseq software (version 0.6.1) based on the ENSEMBL bovine GTF file (version 98) (35).

### Consensus modules detection

The raw count matrix of each dataset was normalized using the voom function of the limma package (version 3.48.3) of R software. The genes with expressions ≥1 count per million reads (CPM) in at least five samples as well as the genes with standard deviations >0.25 across the samples were kept and were scaled (average = 0 and standard deviation = 1) for further analysis. Then, the expression matrix resulted from each dataset was checked for outliers using the adjacency function of the WGCNA R package (version 1.70-3), and the samples with a standardized connectivity score below the threshold of −2.5 were removed (29). In the next step, the filter_by_variance function of the BioNERO R package (version 1.0.4) was applied to remove the genes with low variance across the control samples of each dataset, and the top 10,000 most variable genes, which were common in both datasets, were selected for module detection.

In order to identify consensus modules in control samples of both datasets, the consensus_modules function of the BioNERO R package was used (36). To this end, a signed co-expression network based on Pearson correlation was constructed for each dataset using the exp2gcn function of the BioNERO R package. The Pearson correlation matrix and adjacency matrix were also obtained in control samples as outputs of this function, which were used in the next step. The SFT_fit function of the BioNERO R package was used to pick a power aiming to fit the network to a scale-free topology. In the consensus_modules function, the arguments of correlation method and network type were defined as Pearson and signed network, respectively.

### Preservation analysis

Module preservation analysis across the datasets was performed using the NetRep R package (version 1.2.4) (37). This analysis was performed for each dataset, separately, using modulePreservation function, which uses a permutation test procedure (nPerm = 10,000) on seven module preservation statistics including module coherence (“coherence”), average node contribution (“avg.contrib”), concordance of node contributions (“cor.contrib”), the density of correlation structure (“avg.cor”), concordance of correlation structure (“cor.cor”), average edge weight (“avg.weight”), and concordance of weighted degree (“cor.degree”). “Coherence” calculates the ratio of variance in the module data described by the module”s summary profile vector in the test dataset. “avg.contrib” indicates coherence of the data in the test dataset. “cor.contrib” determines if the module”s summary profile summarizes the data in both datasets similarly. “avg.cor” estimates the intensity of the module correlation on average in the test dataset. “cor.cor” checks the similarity of the correlation heatmaps between the two datasets. “avg.weight” measures the amount of connectivity between nodes in the module on average. “cor.degree” judges whether the nodes that are most strongly connected in the discovery dataset keep this connectivity in the test dataset. In both datasets, normal samples were considered as reference sets, and the infected samples were defined as the test set. For the infected samples from each dataset, the Pearson correlation matrix and adjacency matrix were calculated using the same procedure as for normal samples. Consensus modules were considered preserved if permutation *P*-values for all seven statistics were ≤ 0.01 in both datasets.

### Integrated gene regulatory network construction

To construct an integrated gene regulatory network, lncRNAs and TF genes within a module were identified based on the ENSEMBL bovine GTF file (version 98) and AnimalTFDB database (38), respectively. It is reported that the potential biological function of lncRNAs can be inferred from their nearest neighboring protein-coding genes (cis targets) as well as their co-expressed genes (trans targets) (39, 40). In this regard, all the mRNAs in the module were considered the trans targets of the lncRNAs in that module. On the other hand, the protein-coding genes within a 100 kb window upstream and downstream of each lncRNA were considered cis target genes of those lncRNAs, based on the bovine GTF file.

To further validate these networks, protein-protein interactions (PPIs) among mRNA genes of each module were investigated using the STRING database (version 11.5) (41). This analysis tests whether the number of observed PPIs in each module is significantly more than expected by chance. To enrich the integrated regulatory networks, the obtained PPIs of each module were added to integrated gene regulatory networks. All the obtained interactions among the genes (lncRNAs-mRNAs-TFs) were visualized by Cytoscape software (version 3.8.2) for each module (42).

Highly connected genes (hub genes) were identified using the WGCNA R package. *K*_*ME*_ was used to identify hub genes, and the genes with | *K*_*ME*_| ≥ 0.7 were considered the hub genes. To do this, first, hub genes for each module were identified in each dataset, separately. Then, the overlapped hub genes between two datasets were considered hub genes of consensus modules. This process was applied to find the hub genes of all gene types including mRNAs, lncRNAs and TFs.

Hub genes were also obtained from the PPIs created by STRING. To do this, the Network Analyzer tool of Cytoscape software (version 3.8.2) was used, so that the degree score of each gene was calculated and genes with a degree higher than five were considered hub genes. Then, the common hub genes between the co-expression network and PPIs were identified.

### Functional enrichment analysis

To achieve a deeper comprehension of the biological function of modules, a functional enrichment analysis was performed. The gene ontology (biological process) and KEGG (Kyoto Encyclopedia of Genes and Genomes) pathway enriched in each module were identified using the Enrichr online tool (43). The adjusted *p* ≤ 0.05 (FDR by Benjamini–Hochberg method) was considered to identify significant terms.

## Results

### RNA-Seq data analysis

**Figure 1:**
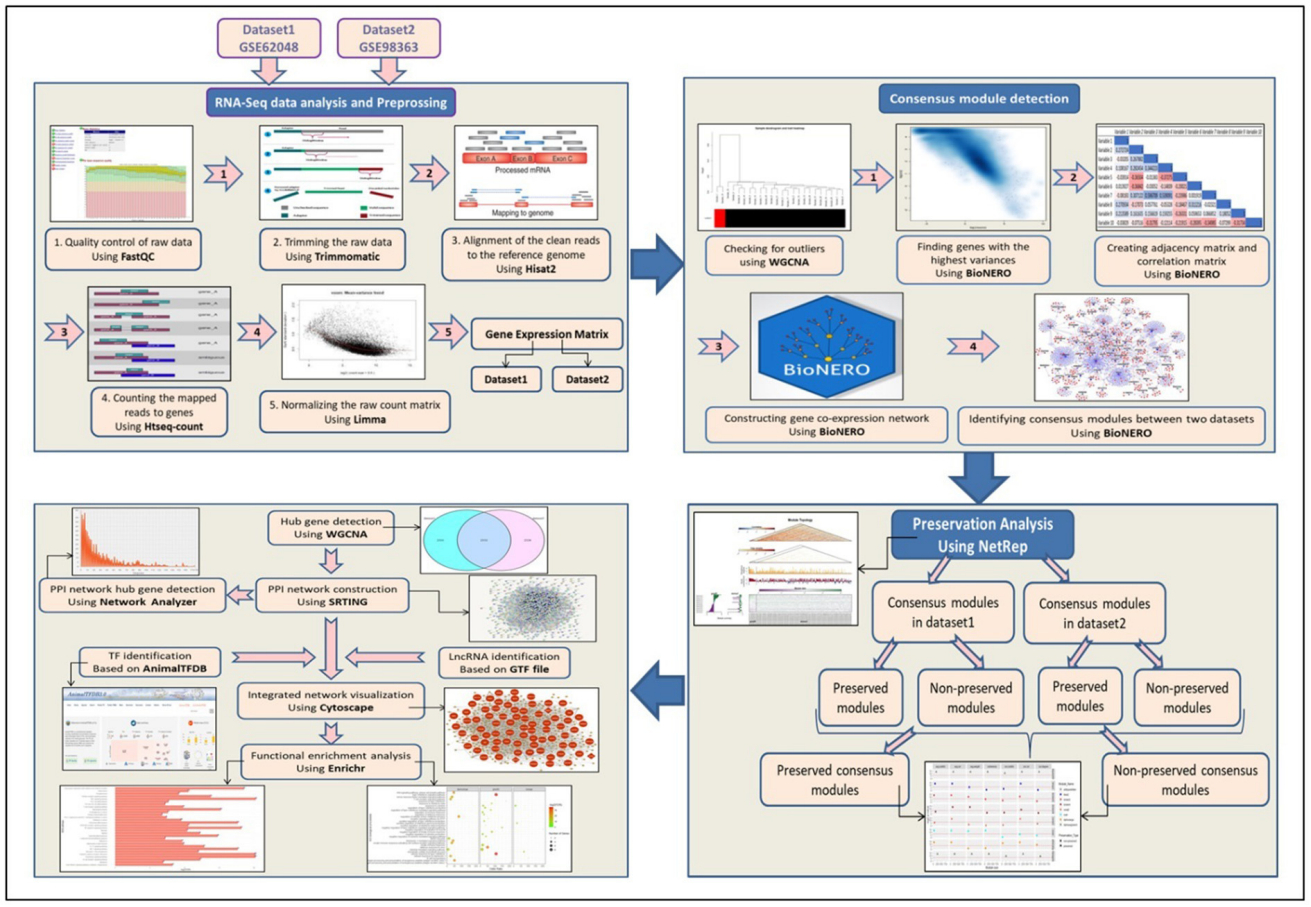
The flow chart of the methodology used in the present study.

The computational analysis pipeline of the proposed method in the present study is shown in Figure 1. The obtained raw reads were 693,703,609 and 4,880,859,643 reads from 35 and 72 samples of the two datasets, respectively. After trimming, 634,958,235 and 4,440,888,983 clean reads were obtained from the two datasets, respectively. The percentages of the aligned clean reads to the reference genome were 76 and 95% in the first and second datasets, respectively. The overview of the RNA-Seq data analysis for both datasets is provided in Supplementary Table 1. A hierarchical cluster analysis of the second dataset showed that two samples (1 and 13 from control samples) were outliers and removed. No outlier samples were detected in the first dataset.

Our filtering pipeline resulted in 11,373 and 11,772 genes in the two datasets, respectively. When the genes with low variance were omitted, 11,013 genes of the first dataset and 11,616 genes of the second dataset remained. Finally, a total of 10,000 overlapped genes from both datasets were used to construct consensus modules across the two datasets.

### Consensus modules and preservation analysis

Soft threshold power beta values of 11 and 12 were determined to achieve a scale-free topology for the construction of the co-expression networks in the two datasets, respectively.

A total of 66 consensus modules were found in normal samples of both datasets, ranging in size from 34 (grey60 module) to 866 genes (firebrick4 module) with a mean module size of 151 genes (Supplementary Table 2).

**Figure 2:**
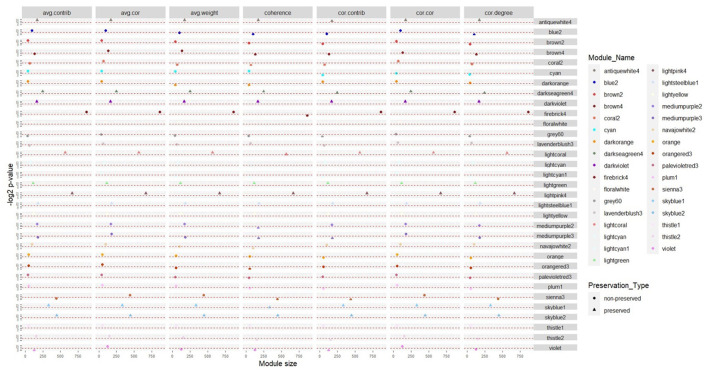
Consensus modules with the same preservation status in the two datasets based on seven module preservation statistics. The modules with –log2 *p*-value in all preservation statistics >6.64 (*p* ≤ 0.01) were preserved, otherwise they were non-preserved.

To assess if the consensus modules were stable in infected samples, preservation analysis was performed. Modules with the same connectivity patterns in both normal and infected samples were considered preserved ones. According to the results of the preservation analysis, in the first dataset, 36 and 30 consensus modules were identified as preserved and non-preserved, respectively (Supplementary Table 3). In the second dataset, 21 consensus modules were preserved, while 45 consensus modules were non-preserved (Supplementary Table 4). The comparison of the preservation status between the two datasets indicated that there were 12 preserved and 21 non-preserved consensus modules common in both datasets (Figure 2). We further focused on the non-preserved consensus modules as they are modules whose connectivity and density are affected by the disease.

### Hub genes identification in non-preserved modules

Overall, 2,532 common hub genes were identified in all consensus modules. Of these, 619 hub genes were members of 21 consensus non-preserved modules. Firebrick4 module owned the highest number of hub genes (227), while the lightcyan1 module had the lowest number (two). The list of the hub genes in the non-preserved consensus modules is presented in Supplementary Table 5.

### Functional enrichment analysis

According to the functional enrichment analysis results, 605 biological processes and 154 KEGG pathways were significantly enriched in the 21 non-preserved consensus modules. Firebrick4 module had the largest number of significantly enriched biological processes terms (160), followed by the sienna3 module (117). In terms of KEGG pathways, 10 modules had significant terms, and the sienna3 module contained the highest number of significantly enriched terms (sixty-four). Brown4, floralwhite, mediumpurple2, plum1, and violet were the modules that showed neither significant biological processes nor significant KEGG pathways. Results of the functional analysis revealed that most of the enriched terms in non-preserved modules were involved in “inflammatory response,” “regulation of interleukin-6 production,” “MAPK cascade,” “regulation of apoptotic process,” “cellular response to type I interferon,” “cytokine-mediated signaling pathway,” “innate immune response,” “response to interferon-gamma,” “T cell receptor signaling pathway” and some relevant genes of these functions were *BLA-DQB, TLR2, NFKB1, IRF1, ATF3, SOCS3, MYC, HMGB1, STAT1*, and *ISG15*.

**Figure 3:**
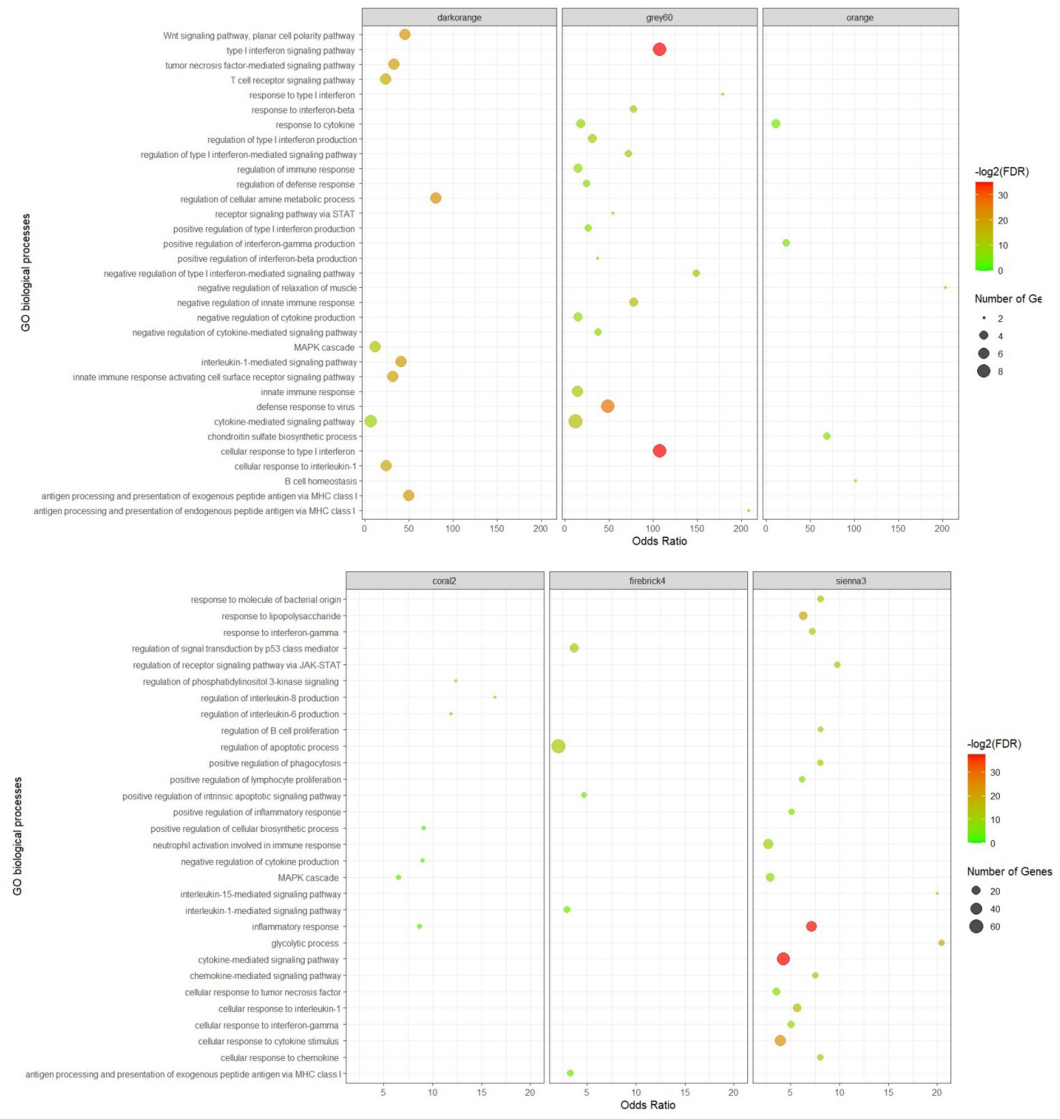
Significant biological processes for the genes in the non-preserved consensus modules. Only top 20 terms were presented for grey60 and sienna3 modules, as they have a lot of significant terms.

**Figure 4:**
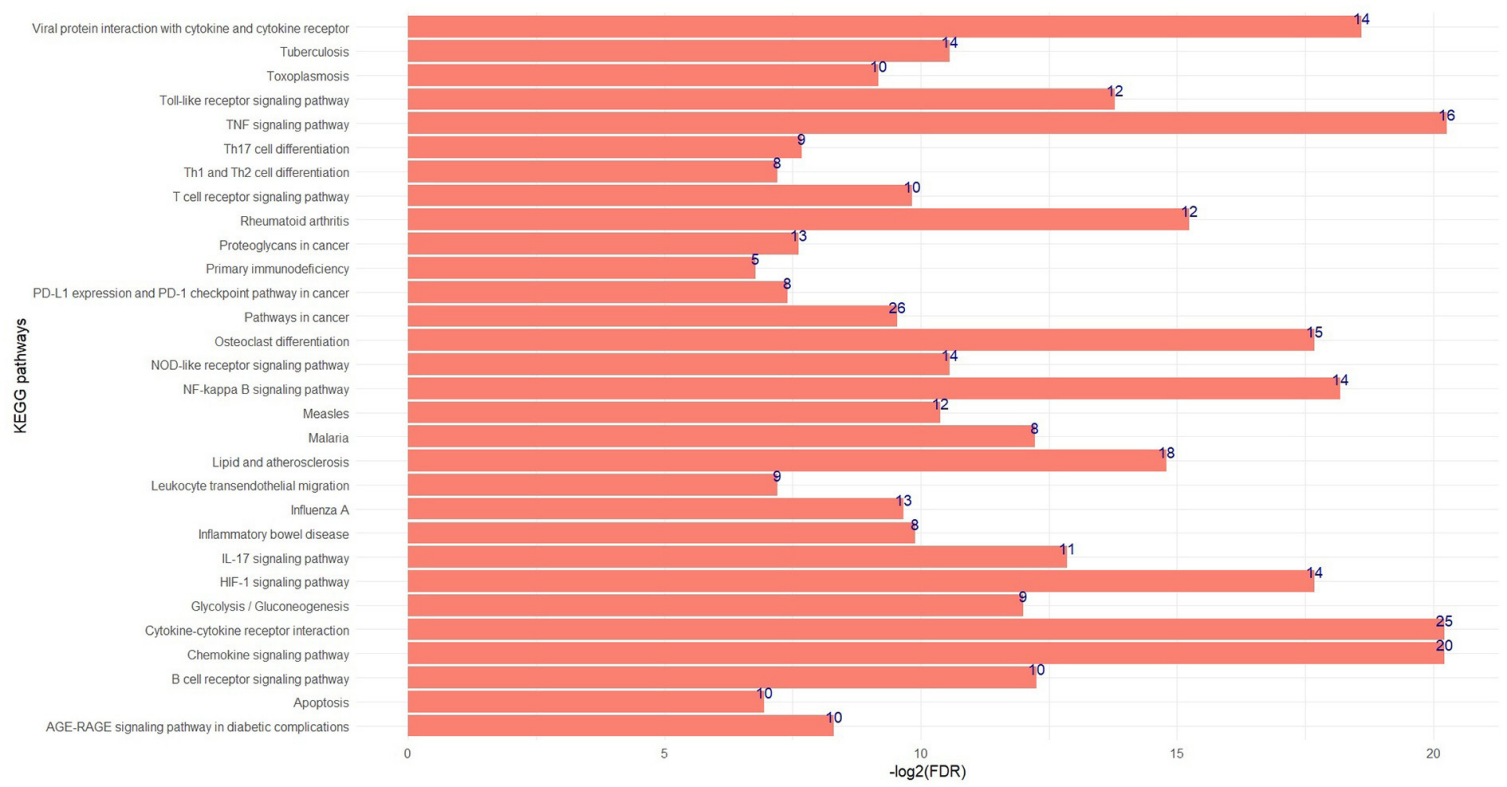
The top significant KEGG pathways in the sienna3 module. The x and y axes indicate –log2 (FDR) and KEGG pathways, respectively. The numbers indicate number of genes enriched by each pathway.

The significant biological processes in some of the non-preserved modules are depicted in Figure 3, and the most significant KEGG pathways in the sienna3 module are shown in Figure 4. The complete list of the functional enrichment analysis for the non-preserved consensus modules is available in Supplementary Tables 6, 7. It is well reported that disease-related modules should correspond well to biological functions associated with diseases (26–28). Hence, a non-preserved consensus module was considered as JD-related if its gene members were significantly enriched for some of the functional terms associated with disease such as “inflammatory response,” “interleukin-1-mediated signaling pathway,” “type I interferon signaling pathway,” “cytokine-mediated signaling pathway,” “regulation of interferon-beta production,” and “response to interferon-gamma.” Eight out of 21 modules were enriched for at least one of these terms, and the sienna3 module because of owning the highest number of terms related to JD was considered as the most important non-preserved module.

**Figure 5:**
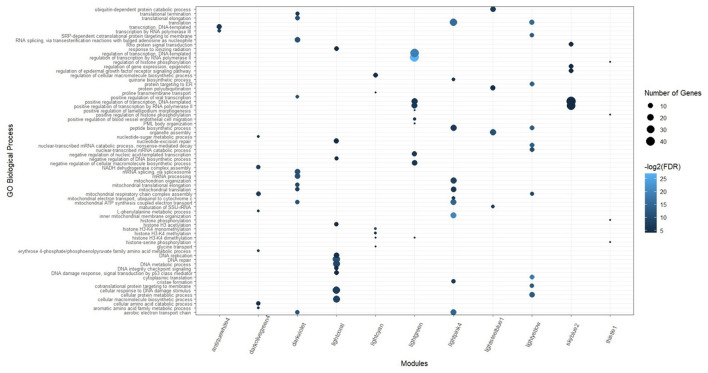
Significant biological processes for the genes in the preserved consensus modules. Only top 10 terms were presented for darkviolet, lightpink4, and lightyellow modules, as they have a lot of significant terms.

Based on the KEGG and gene ontology analysis, 151 biological processes and 61 pathways were significantly enriched in the 12 preserved consensus modules. The most significant enriched terms of biological processes and KEGG pathways belonged to lightpink4 (forty-six) and darkviolet (sixteen) modules, respectively. Skyblue1 was the only module without any significant biological processes. While, there were several modules with no significant KEGG pathways including antiquewhite4, lightgreen, lightsteelblue1, and thistle1. The highly enriched biological processes in the preserved modules were “mRNA processing,” “RNA splicing,” “transcription by RNA polymerase III,” “translational elongation,” and “cytoplasmic translation.” The results of the functional analysis in the preserved consensus modules are illustrated in Figure 5. The complete list of the functional enrichment analysis for these modules is provided in Supplementary Tables 8, 9.

### Integrated gene regulatory networks

Out of 21 non-preserved consensus modules, 34 lncRNA and 152 TF genes were identified in 12 and 19 modules, respectively. The largest number of lncRNAs (nine) and TFs (sixty-three) were found in the firebrick4 module. Five non-preserved modules had hub lncRNAs including brown4 (two), mediumpurple2 (one), navajowhite2 (one), plum1 (one), and sienna3 (one). Hub TFs were also found in nine non-preserved modules comprising brown4 (one), firebrick4 (nine), floralwhite (one), lavenderblush3 (one), mediumpurple3 (three), navajowhite2 (three), orange (one), sienna3 (three), and thistle2 (three). The complete list of the identified TFs and lncRNAs in the non-preserved consensus modules are presented in Supplementary Tables 10, 11, respectively. For all of the lncRNAs in 12 modules, cis targets were identified (Supplementary Table 12). Of these, trans targets of four lncRNAs were identified in the same modules (brown4, sienna3, and thistle2) (Supplementary Table 13). It is noteworthy that these lncRNAs and their targets in brown4 and sienna3 modules were hub genes too. These results suggest a positive feedback loop among the regulators and the other module members.

Then, the enrichment in PPIs for each non-preserved consensus module was assessed using the STRING database. PPIs of 17 modules were significant, which indicates that the number of observed PPIs in each module is significantly more than expected by chance. However, four modules including coral2, lightcyan1, plum1, and violet did not have significant PPIs. The number of identified nodes and edges for each module are provided in Supplementary Table 14. Totally 1,063 hub genes (degree > 5) were explored in PPI networks of the non-preserved consensus modules. Firebrick4 module possessed the largest number (652), and blue2 and plum1 modules each with one hub gene showed the lowest numbers. Lightcyan1 module was the only module without a hub gene in the PPIs network. Also, 283 hub genes were detected in both PPIs and WGCNA in the non-preserved consensus modules. The list of the PPIs network hub genes in the non-preserved consensus modules is presented in Supplementary Table 15.

**Figure 6:**
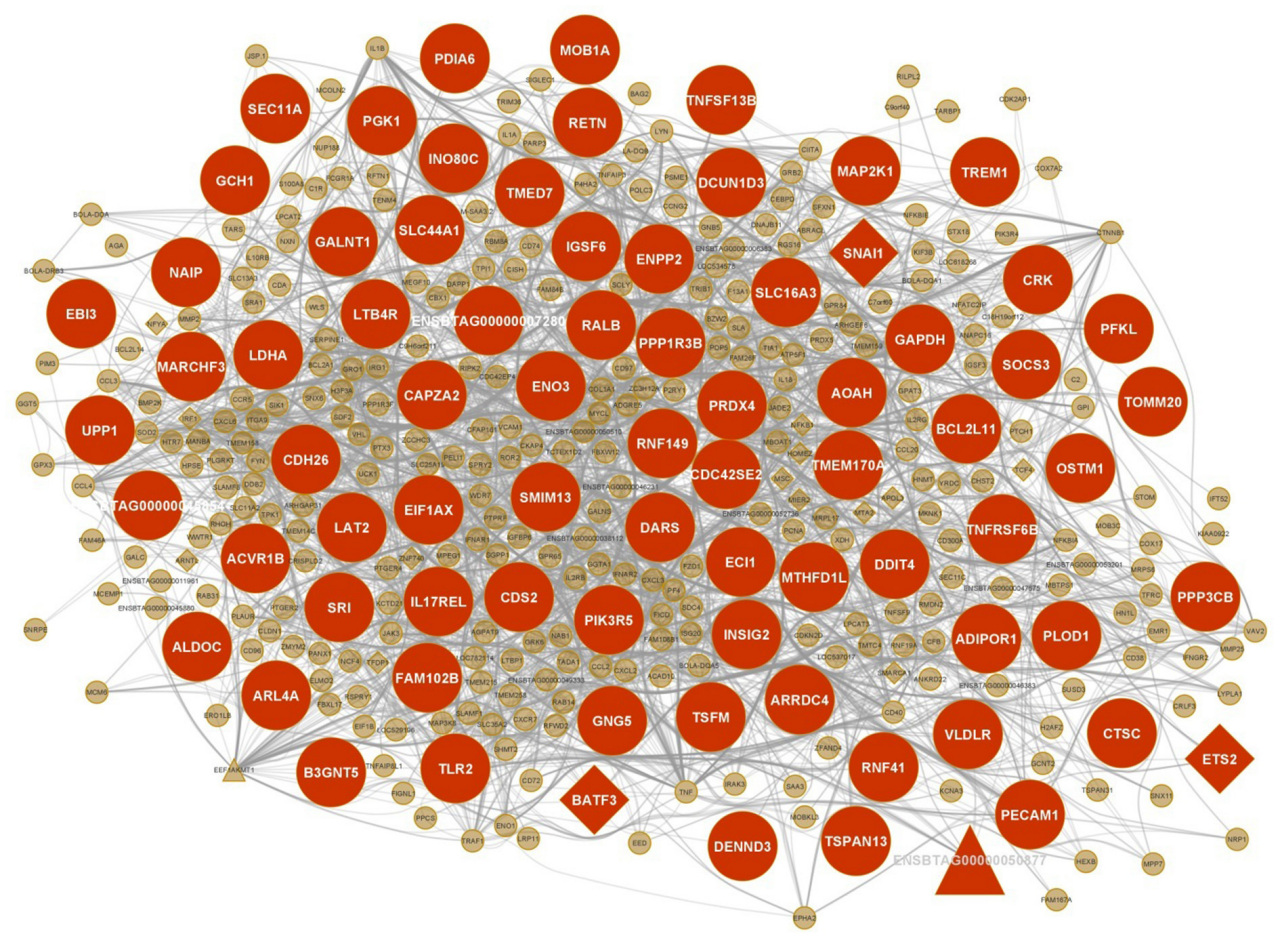
Integrated regulatory network of sienna3 module. Each node represents a gene and each edge represents the interaction between genes. mRNAs, lncRNAs and TFs are indicated with circles, triangles and diamond shapes, respectively, and the larger shapes represent hub genes.

The integrated gene regulatory network of one of the most important non-preserved consensus modules (sienna3 module) including mRNAs, lncRNAs, and TFs is shown in Figure 6.

## Discussion

It is undeniable that JD causes economic losses in dairy farms; however, the specific pathogenesis of the disease and the molecular mechanism of its regulation are still unclear. Therefore, providing new procedures in order to broaden our knowledge of this complex disease can help identify networks of genes involved in JD pathogenesis. Since non-reproducibility of the results is a major problem in biological research and all the studies that have already been performed associated with JD focused only on one dataset, in the present study, a computational pipeline was proposed to authenticate the repeatability of the obtained results in two datasets. Here, consensus modules were defined as the modules comprised of genes tightly co-expressed in normal samples of both datasets and exhibit a degree of reproducibility between the two networks. Here, two datasets with different time points and replications per time point were applied to find the consensus modules. In this context, it is reported that more stable modules are likely to replicate in studies with different sample sizes or other technical features. Moreover, it is demonstrated that more stable modules are more corresponded to well-characterized biological functions (44). Hence, the identified consensus modules in the present study can be considered stable modules that can be efficiently annotated across the well-known biological functions. There are other studies that identified consensus modules related to different traits. For instance, Yuan et al. identified eight consensus gene co-expression modules associated with abdominal fat deposition across multiple broiler lines. They also found genes and pathways related to the trait in these modules (45). On the other hand, the non-preserved consensus modules are the consensus modules whose connectivity and density are altered in infected samples of both datasets. We hypothesized that the non-preserved consensus modules would guide us to identify those gene sets that are highly involved in biological processes relevant to the disease. Moreover, focusing on the hub genes in these modules enables us to identify genes that may play central roles in JD progression with more confidence.

Enrichment analysis revealed that in the preserved modules, genes were mainly enriched in the general processes of cells such as “transcription,” “mRNA processing,” “mRNA splicing,” “DNA replication,” “translation,” and “translational termination.” On the other hand, the non-preserved modules were mainly enriched in biological functions related to the immune system, which is largely affected by MAP infection and can be associated with JD progression. Out of 21 non-preserved consensus modules, eight modules showed significantly enriched terms associated with JD, some of which are discussed as follows. These modules showed significant PPIs, except for the coral2 module, and there were many common hub genes between the co-expression network and the PPIs network in these modules. Some of the hub genes and also biological processes in these modules, mentioned in the following, were in accordance with the results of our previous work on the construction of integrated networks related to MAP infection; nevertheless, detecting and analyzing consensus modules using the new pipeline allowed us to identify other important genes and processes possibly associated with JD more confidently.

Sienna3 module was considered to be the most important module since functional enrichment analysis indicated that this module was enriched in the highest number of biological processes related to JD (30 terms) including “cytokine-mediated signaling pathway,” “inflammatory response,” “cellular response to interleukin-1,” “response to interferon-gamma,” “neutrophil activation involved in immune response,” “regulation of B cell proliferation,” and “positive regulation of phagocytosis” (Supplementary Table 6). Furthermore, the sienna3 module also showed several significant KEGG pathways associated with JD such as “TNF signaling pathway,” “Chemokine signaling pathway,” “Toll-like receptor signaling pathway,” “IL-17 signaling pathway,” “B cell receptor signaling pathway,” “T cell receptor signaling pathway,” and “Th1 and Th2 cell differentiation” (Supplementary Table 7). From the enriched biological processes and pathways in the sienna3 module, “neutrophil activation” and “T cell differentiation” were in accordance with the identified pathways in the non-preserved modules in our previous work on MAP infection (28). MAP infection activates various immune-related pathways most of them were significantly enriched in the sienna3 module. In response to MAP infection, macrophages secrete pro-inflammatory cytokines such as interleukin-1, TNF, and interferon-gamma (46, 47). Cytokines play a crucial role in host immunity through activating cells to kill pathogens or setting responses to control the disease. IFN-γ activates the antimicrobial mechanisms of the macrophage in order to destroy pathogens. It also contributes to Th1 differentiation, T-cell activation, and dendritic cells maturation. Numerous functions of IFN-γ are indicative of its role in the control of MAP infection (47). IL-1 is essential in both protective immunity and MAP survival (31). Pro-inflammatory cytokines potentially cause the formation of lesions in the ileal tissues of infected animals. These lesions actively hold infection, but clinical signs are not usually apparent (31, 48). Th-cell differentiation is an important pathway during JD development. The initial immune response of MAP-infected cattle in the subclinical stage is Th1; however, with the progression of disease in the late subclinical stage, there is a shift to Th2-mediated humoral response. Since Th2 is a non-protective response and cannot control intracellular infections, animals enter the clinical phase of the disease (49).

A comprehensive literature review showed that many hub genes and some TFs of the sienna3 module are reported to be associated with JD and MAP infection including *BLA-DQB, TLR2, RNF149, ADIPOR1, AQP9, TREM1, GCH1, PIK3R5, SOCS3, NFKB1, IRF1*, and *CDH26*. Some of these genes including *TREM1, BLA-DQB, IRF1, ADIPOR1*, and *CDH26* were also reported associated with MAP infection in our previous study (28). *BLA-DQB* hub gene is one of the MHC II genes, which is also significantly inhibited in MAP-exposed cattle (50). Since MHC class II antigens have an important role in the immune response, downregulation of the MHC class II during MAP infection may disrupt the activation of an immune response against infecting pathogens. The expression of MHC class II genes has been reduced in MAP-exposed cows. The inhibition of these genes may influence the host's ability to deliver MAP fragments to CD4+ T lymphocytes (50). *TLR2* is a member of Toll-like receptors (TLRs). TLRs are the pattern recognition receptors that bind pathogen-associated molecular patterns (PAMPs) and are intrinsically involved in activating both the innate and adaptive immune response mechanisms. *TLR2* is especially involved in the early recognition of mycobacterial antigens (51). In fact, *TLR2* plays an important role in phagosome trafficking and antimicrobial responses in MAP-infected bovine phagocytes (52). The site of *TLR2* expression in cattle is myelomonocytic cells, and its expression on bovine macrophages is eight times higher than that on dendritic cells (53, 54). Since the target cells of MAP to proliferate and survive are bovine macrophages, *TLR2* has a key role in JD (52). Koets et al. deduced that cows with a susceptible *TLR2* haplotype may experience the clinical phase of JD at a younger age, or more severe, due to deficient innate and subsequent cell-mediated immune responses (19). Previous studies on JD found that the presence of single nucleotide polymorphisms (SNPs) in this gene were significantly associated with this disease (19, 24). Moreover, Mucha et al. found seven missense mutations in *TLR2* gene associated with increased MAP susceptibility in Holstein cows (55). Ruiz-Larrañaga et al. confirmed the role of *TLR2* gene in susceptibility to MAP infection (56). In another study, *TLR2* was suggested to be a biomarker for MAP-infection in domestic animals (57). *RNF149* is located within a QTL associated with humoral response to MAP (20). It has also been differentially expressed in infected monocyte-derived macrophages of cows (16). In addition, studies have shown that SNPs in *ADIPOR1* are significantly associated with MAP infection status (22, 58). Statistically significant SNPs are reported in *AQP9*, which are associated with MAP resistance/susceptibility (21, 22). Also, *AQP9* has been identified to be a differentially expressed gene at different times after MAP infection (59). *TREM1* is reported as a positional candidate gene in QTLs associated with MAP infection (60). *TREM1* strengthens the inflammatory response to invading microbes and has a key role in protective innate immunity during MAP infection (17, 61). This gene has been downregulated in MAP-infected animals, and it seems to be as a result of MAP invasion, which causes the host cell to suppress the expression of surface receptors such as TLRs and MHC class II molecules (required for pathogen recognition) (62). *GCH1* is another hub gene of the sienna3 module with a significant SNP associated with MAP infection (22). The significant association of *PIK3R5* hub gene with susceptibility/resistance to JD was also reported (63). This gene is involved in TNF signaling and chemokine signaling pathways (64). The increased expression of *SOCS3* hub gene in response to MAP infection has been observed in several studies (10, 17, 65). This gene is an anti-inflammatory cytokine, and it is suggested that its upregulation participates in the inhibition of the JAK-STAT pathway. In fact, it is a strategy that MAP uses to survive inside the macrophage (65). *NFKB1* is an important TF regulating many immune function genes and directly participates in IL-1 activation (66). *NFKB1* encodes proteins belonging to the mitogen-activated protein kinase (MAPK) signaling cascade so that these proteins trigger the downstream cellular responses upon the recognition of mycobacterial pathogen-associated molecular patterns (PAMPs) (8). *NFKB1* was upregulated in the infected monocyte-derived macrophages (11, 16, 17). It is also revealed that this gene is significantly expressed during the late phase of infection (67). *NFKB1* is recognized to be associated with MAP infection in a SNP-based gene set enrichment analysis (68). *IRF1* is a member of the interferon regulatory TF family and an activator of interferon alpha and beta transcription. It was upregulated across different time points post-infection (11). *IRF1* was found in genomic regions having SNPs associated with MAP infection (21, 25). *IRF1* significantly contributes to many immune responses including the Type 1 (Th1) cell-mediated immune response. Cell-mediated immunity is an important host defense mechanism against intracellular pathogens including MAP (69). In the sienna3 module, *CDH26* gene was found as both cis and trans target of lncRNA ENSBTAG00000050877. Surprisingly, both lncRNA and its target were hub genes. *CDH26* encodes a member of the cadherin protein family. The protein is expressed in gastrointestinal epithelial cells and may be upregulated during allergic inflammation. In addition, this protein interacts with alpha integrins and may also be involved in leukocyte migration and adhesion (70). Among the investigated hub genes of this module, several genes were also identified as hubs in the PPIs network including *TLR2, ADIPOR1, TREM1, GCH1, PIK3R5, SOCS3*, and *CDH26*. Moreover, there were several other genes such as *NAIP, RETN, CRK, PIK3R5, PRDX4, LTB4R, PFKL, TNFSF13B, PECAM1, ALDOC, GAPDH, AOAH, BCL2L11*, and *MAP2K1* that were hubs in both WGCNA and PPIs network and according to functional enrichment analysis were involved in immune-related processes and JD. Overall, these findings may provide a basis for further studies on the role of the sienna3 module's genes in the progression of JD.

Genes from the firebrick4 module showed enrichment for “positive regulation of intrinsic apoptotic signaling pathway,” “antigen processing and presentation of exogenous peptide antigen via MHC class I,” and “interleukin-1-mediated signaling pathway,” which are related to JD. “Regulation of intrinsic apoptotic signaling pathway” was also one of the enriched pathways in the non-preserved modules in our previous work (28). Mycobacteria induce apoptosis in macrophages and release apoptotic vesicles that deliver mycobacterial antigens to uninfected antigen-presenting cells. Pro-apoptotic signals probably cause the removal of pathogens, while anti-apoptotic signals can either eliminate the pathogen and control the infection by improving antigen presentation to T cells or suppress the immune response and enable pathogen survival (11, 71, 72). MHC class I molecules, through which antigen presentation occurs, are involved in T cytotoxic cell recognition in infected cells (10). It is also suggested that MAP subverts apoptosis to prevent antigen presentation and escape the host immune response. So MAP survival in macrophages leads to clinical disease (31, 73). Numerous studies referred to the hub genes and TFs of this module as participants of JD pathogenesis including *SMARCA5, RHOA, GTF2A2, KTN1, LEO1, USP8, EIF4E, MYC, ATF3, GTF2I*, and *HMGB1*. *SMARCA5* is found to be linked to a SNP related to the antibody response to MAP in cattle (23). *RHOA* is a small GTPase protein located in intestinal epithelial cells and is linked to the process by which MAP crosses the intestinal barrier (74). *GTF2A2, KTN1, LEO1*, and *USP8* are reported as candidate genes with SNPs significantly associated with JD (21, 22). *EIF4E* is also associated with JD according to other studies (10, 75). This gene plays a role in the immune system and cytokine signaling pathways (10). *MYC* is a TF in this module, and its expression has been increased after MAP infection in cows (17). c-Myc has a key role in macrophage differentiation (76, 77). *ATF3* is another TF gene that uses an epigenetics mechanism to bind and suppress both its own promoter and other *ATF3* target genes (78). This gene has been induced by MAP infection, and its expression has been sustained throughout the infection period. Therefore, it is suggested that *ATF3* repressive regulation may suppress pro-inflammatory chemokine production (17, 79). It is also demonstrated that *ATF3* has an important role in modulating IFN responses in macrophages (80). *GTF2I TF* gene is identified as a candidate gene to be relevant to MAP susceptibility (23). This gene is involved in many important cellular processes including apoptosis, neuro-active ligand-receptor interaction, calcium signaling pathway, cytokine–cytokine receptor interaction ribosomal pathway, gap junction, and adherens junction (81). Since *GTF2I* regulates genes related to defense mechanisms and may be linked with apoptosis in macrophages, it can be suggestive of its role in the pathogenesis and persistence of mycobacteria in macrophages, which is associated with MAP infection (82). *GTF2I* is also known as one of the genes related to MAP infection in our previous paper (28). *HMGB1* is a TF gene and plays a part in the regulation of the immune response (83). This gene is involved in the repressed state of phagocytosis for MAP condition in the late stage (84). It is reported that the expression of *HMGB1* has been decreased in JD-infected cows (18). All the hub genes discussed in this module were also hub genes in the PPIs network, which reinforce their potential functions in JD. Our results suggested that the identified regulatory interactions of the firebrick4 module's members might contribute to understanding the potential molecular mechanisms underlying JD development.

The most significant biological processes associated with JD in the coral2 module were “inflammatory response,” “regulation of interleukin-8 production,” “positive regulation of cellular biosynthetic process,” “negative regulation of cytokine production,” “regulation of phosphatidylinositol 3-kinase signaling,” “regulation of interleukin-6 production,” and “MAPK cascade.” From these, the mitogen-activated protein kinase (MAPK) pathway is one of the key pathways involved in the host response to MAP (11). MAPK cascade activates downstream cellular responses once the mycobacterial pathogen is recognized by cell surface pathogen recognition receptors (8). MAPKs also play important roles in the regulation of the expression of genes encoding inflammatory chemokine and cytokines through activation of several transcription factors (85, 86). *TCF12* is a TF gene in the coral2 module with a SNP within genomic regions associated with MAP resistance/susceptibility (21). *PIK3CB* was detected as the hub gene in both co-expression and PPI networks. This gene may be directly involved in JD pathogenesis, because of its role in MAPK cascade and regulation of phosphatidylinositol 3-kinase signaling.

The genes in the grey60 module were enriched in several important biological processes related to JD such as “cellular response to type I interferon,” “negative regulation of innate immune response,” “cytokine-mediated signaling pathway,” “negative regulation of type I interferon production,” “response to interferon-beta,” and “response to cytokine.” Type I interferons mainly include IFNα and IFNβ. It is reported that IFNα/β may have two contradictory roles in bacterial infection. On the one hand, they may protect the host against infection by upregulating antimicrobial effectors like pro-inflammatory cytokines. On the other hand, they may destroy the host response to bacteria through different immune functions such as evoking IL-10 and IL-1 receptor antagonist production, inhibiting pro-inflammatory cytokine production, inducing apoptosis, and limiting host responses to IFN-γ. Type I IFNs have detrimental effects on intracellular bacteria. Although the mechanisms are not clear, it is suggested that IFNα/β suppress the production of host-protective cytokines in mycobacterial infections (87). *ISG15*, as a hub gene of this module, is believed to be related to JD because of having a significant SNP associated with MAP infection (22). This gene is linked to a defense response to invading pathogens (88). *STAT1* is a TF gene of the grey60 module and indirectly induces an immune response. Once *STAT1* translocates into the nucleus, activates transcription of IFN-γ-inducible genes (89). IFN-γ acts primarily through the regulation of gene expression to induce macrophages to kill intracellular pathogens (65). Furthermore, *STAT1* was reported to be differentially expressed in JD-positive cows (90). Both *ISG15* and *STAT1* were mentioned as JD-related genes in our previous work on MAP infection (28). *ISG15* and *IFI27* were identified as hub genes in both WGCNA and STRING networks. These genes significantly participated in many biological processes related to the immune system based on the functional enrichment analysis.

In terms of functional analysis, the orange module was associated with immune system biological processes including “B cell homeostasis,” “positive regulation of interferon-gamma production,” and “response to cytokine.” The role of cytokines and IFN-γ in MAP infection was mentioned earlier. It is noteworthy that the elevated level of IFN-γ is considered as an immunological characteristic of the subclinical stage of JD when macrophages are activated by IFN-γ to kill the bacteria (91). *ADAM10* is a hub gene of the orange module, and it is considered a candidate gene associated with MAP infection (21, 22). *CASP3*, as another hub gene of the orange module, is a protease and takes part in apoptosis (92). Several studies reported that *CASP3* expression is significantly affected by MAP infection (15, 16). This gene was a common hub gene in both predicted networks, and the functional analysis showed that it plays a role in the process of response to cytokine.

In the blue2 module, the most significant biological processes were “regulation of autophagy,” “autophagosome organization,” and “autophagosome assembly.” The role of autophagy in MAP infection has been pointed out in several studies (11, 12, 93). In fact, the main defense mechanism against MAP is destroying macrophages through autophagy and apoptosis (88). It is supposed that autophagy acts as a compensatory mechanism to present intracellular MAP antigens at the time of decreased antigen presentation by MHC-1 (12). Autophagosomes are double-membrane vesicles that contain host cell cytosolic components. In the process of autophagy, autophagosomes fused with lysosomes to degrade their contents (11). *TTC7A* is a hub gene of this module and was reported as a candidate gene with known SNPs associated with JD (21, 22).

## Conclusion

The pipeline presented in this study can pave the way to profit more valid results since it enables us to identify consensus modules, which are non-preserved in infected treatment. In other words, it is a new approach for validation of the results. The identified non-preserved consensus modules in the two datasets are helpful to enhance our knowledge and understanding of molecular mechanisms connected with JD. Among these modules, the ones whose biological functions as well as genes were associated with JD are of special importance, so that they are sources of genes that can be prospective candidates for diagnosis and prognosis of JD. It is worth considering that, because of high connectedness and regulatory roles, hub, TF, or lncRNA genes of the non-preserved modules are more critical in discovering JD pathogenesis and can be considered new candidate biomarkers. The enrichment analysis results demonstrated the roles of these genes in biological processes and pathways linked to the immune system and JD development. It is noteworthy that some of the biological processes and pathways as well as genes reported in our previous paper were found in the non-preserved consensus modules; however, we could achieve more comprehensive and reliable results with the aid of the new pipeline in the present study.

## Data availability statement

Publicly available datasets from Gene Expression Omnibus (GEO) database at the National Center for Biotechnology Information (NCBI) were analyzed in this study. The datasets can be found under GSE62048 (https://www.ncbi.nlm.nih.gov/geo/query/acc.cgi?acc=GSE62048) and GSE98363 (https://www.ncbi.nlm.nih.gov/geo/query/acc.cgi?acc=GSE98363) accession numbers.

## Author contributions

MB conceived the ideas and designed the study. MH analyzed and interpreted the data and wrote the main manuscript text. AP supervised this work and provided financial support for the project. MB, AP, and FD reviewed and edited the manuscript. All authors read and approved the final manuscript.

## Funding

This research is supported by Isfahan University of Technology.

## Conflict of Interest

The authors declare that the research was conducted in the absence of any commercial or financial relationships that could be construed as a potential conflict of interest.

## Publisher's note

All claims expressed in this article are solely those of the authors and do not necessarily represent those of their affiliated organizations, or those of the publisher, the editors and the reviewers. Any product that may be evaluated in this article, or claim that may be made by its manufacturer, is not guaranteed or endorsed by the publisher.

## Abbreviations

JD: Johne's disease
KEGG: Kyoto Encyclopedia of Genes and Genomes
lncRNAs: long non-coding RNAs
MAP: mycobacterium avium subsp. paratuberculosis
PPIs: protein-protein interactions
SNP: single nucleotide polymorphism
TFs: transcription factors
WGCNA: weighted gene co-expression network analysis.
